# Metacognitive Therapy for Depression Reduces Interpersonal Problems: Results From a Randomized Controlled Trial

**DOI:** 10.3389/fpsyg.2018.01415

**Published:** 2018-08-07

**Authors:** Eivind R. Strand, Roger Hagen, Odin Hjemdal, Leif E. O. Kennair, Stian Solem

**Affiliations:** Department of Psychology, Norwegian University of Science and Technology, Trondheim, Norway

**Keywords:** depression, metacognitive therapy, interpersonal problems, RCT, psychotherapy

## Abstract

Interpersonal problems are significantly elevated in patients with depression. Metacognitive therapy (MCT) for depression does not address interpersonal problems but is associated with large reduction in depressive symptoms. The main aim of the current study was to explore whether MCT leads to improvements in interpersonal problems in patients with depression. The study was a waitlist controlled trial and assessments took place at pre- and post-treatment as well as 6-month follow-up. At pre-treatment, the sample had more interpersonal problems compared to samples from other studies of psychiatric outpatients. MCT was associated with large reductions in interpersonal problems. Level of interpersonal problems were not related to poorer treatment response. MCT, which does not directly target interpersonal problems, worked well for patients with depression and interpersonal problems. Future research should compare MCT with other evidence-based treatments for patients with depression and interpersonal problems.

## Introduction

Interpersonal problems are common among patients with psychiatric disorders and especially patients with depression (Barrett and Barber, 2007; Bjerke et al., 2011). Many patients show interpersonal rumination involving analyzing and anticipating distress in relational situations that could involve being offended, criticized, and humiliated (Ottavi et al., 2016). Treatment addressing such repetitive thinking in depression could be potentially beneficial and reduce interpersonal problems. Interpersonal problems are defined as unremitting difficulties experienced by individuals in their social relationships (Horowitz et al., 1988, 1993). For people struggling with major depressive disorder (MDD), interpersonal problems such as social difficulties and poor peer relationships seem to be present from early age (Lewinsohn et al., 2003). Furthermore, interpersonal domains of distress have been found to predict recurrence of MDD over and above well-recognized depression risk factors such as dysfunctional cognitions and personality disorder symptoms in emerging adults (Sheets and Craighead, 2014).

Studies of people with depression, using the Inventory of Interpersonal Problems (IIP; Horowitz et al., 1993), have suggested a socially avoidant interpersonal style in people with depression (Barrett and Barber, 2007; Renner et al., 2012). This could lead to depressed patients being isolated and bereaved of potential resources of their social environment, such as social support that has been associated with being more likely to achieve complete satisfactory mental health after suffering from depression (Fuller-Thomson et al., 2016).

Previous research indicates an array of interpersonal factors as inherent to depression. These include insecure attachment orientations (Bifulco et al., 2006), excessive reassurance seeking (Joiner and Metalsky, 2001), passivity, and being withdrawn (Allan and Gilbert, 1997). There could also be deficiencies in interpersonal style in-group interactions (Youngren and Lewinsohn, 1980). Additionally, interpersonal processes such as excessive reassurance seeking and negative feedback seeking as responses to negative affect could be important interpersonal processes leading to and maintaining depression (Evraire and Dozois, 2011). Interpersonal characteristics have also been associated with treatment outcomes (Blatt et al., 1996) where a friendly and submissive style is associated with better therapeutic alliances and therapy outcomes, whereas a dominant and hostile style is associated with poorer outcomes (Muran et al., 1994; Gurtman, 1996; Borkovec et al., 2002). However, other studies have found non-significant associations between pre-treatment IIP and treatment outcome (e.g., Hoyer et al., 2014; Ung et al., 2017). This is also supported by a recent meta-analysis showing that some studies find significant associations while others do not (McFarquhar et al., 2018). This meta-analysis also concluded that there is a notable lack of reports on interpersonal problems in depression trials and that treatment effect size when using the IIP (in brief psychotherapy) was *g* = 0.74 (McFarquhar et al., 2018).

Although there seems to be a link between interpersonal problems and depression, there seems to be a lack of adequate theoretical models and frameworks for understanding the above-mentioned processes (Evraire and Dozois, 2011). Interpersonal theories have sought to understand underlying interpersonal dynamics hypothesized to be causal and maintaining factors in psychological disorders (Horowitz et al., 1993). Proponents of an interpersonal approach of depression have argued that “*the strongest implication of the interpersonal approach is that depression not only has interpersonal features and consequences but also is fundamentally interpersonal in nature*” (Joiner et al., 1999, p. 7).

Interpersonal psychotherapy (IPT; Weissman et al., 2000) for depression deals with resolving interpersonal problems. Furthermore, IPT understands interpersonal issues to be a central factor in the genesis and maintenance of psychological symptoms. Unlike IPT, metacognitive therapy (MCT: Wells, 2000) for depression does not focus on interpersonal problems. MCT is based on the Self-Regulatory Executive Function model (S-REF model, Wells and Matthews, 1994, 1996), which provides a theoretical framework for understanding the initiation and maintenance of emotional disorders. Emotional disorders result, according to metacognitive theory, from an inflexible and maladaptive thinking style, termed the cognitive attentional syndrome (CAS: Wells and Matthews, 1994, 1996; Wells, 2000). The CAS consists of worry, rumination, threat monitoring, and dysfunctional coping strategies. MCT considers rumination to be a cognitive coping strategy characterized by perseverative dwelling on thoughts, feelings, and previous events.

Cognitive attentional syndrome strategies, such as rumination and worry, are in turn hypothesized to be driven by metacognitive knowledge rather than external factors. In particular, negative metacognitive beliefs about the uncontrollability and danger of thinking are likely to hinder awareness of executive control such as attentional flexibility, thereby resulting in persistence of the CAS and emotional distress (Wells and Matthews, 1994; Wells, 2000). According to the metacognitive model, the activation of the CAS can have interpersonal consequences by enhancing emotional distress or by the selection of maladaptive coping strategies such as avoidance or drinking alcohol.

Metacognitive therapy aims to enhance self-regulatory skills and predict that modifying underlying metacognitive beliefs and replacing the CAS with adaptive coping will enhance self-regulatory capacity. This should be beneficial for dealing with interpersonal issues and external stressors. Interpersonal problems and distress are therefore only addressed indirectly in MCT when linked to CAS activity, to socialize the patient to the model through modifying erroneous metacognitive beliefs and enable flexible executive control. Recently, the theoretical metacognitive model for depression was supported, as metacognition and rumination were found to explain a significant amount of variance (51%) in depressive symptoms (Solem et al., 2016). Results also suggest that MCT could be an efficient treatment for depression (e.g., Hagen et al., 2017; Hjemdal et al., 2017). However, how MCT affects interpersonal problems and *vice versa* is unexplored.

The main hypotheses of the current study were therefore as follows:

1. Interpersonal problems are present in patients with depression and correlated with depression severity.
2. MCT is an effective treatment for interpersonal problems in patients with depression.
3. Interpersonal problems are correlated with poorer treatment response.

## Materials and Methods

### Participants

The intent to treat sample consisted of 39 patients with a diagnosis of depression. The sample had a mean age of 33.7 (*SD* = 10.4, range = 18–54) and 23 of 39 were women. The mean age for the debut of depression was 26.2 (*SD* = 11.7). Approximately half of the sample (51.3%) was married/cohabitant and 84.6% were of Norwegian ethnicity. With respect to employment, 30.8% were in full-time jobs, and 17.9% were full-time students. The remaining participants received social or disability benefits. A total of 38.5% had a college/university degree. Only 7.7% used SSRIs, while 59% had received treatment previously for their depression. Thirty patients had seen their general practitioner because of depression, 9 had been medicated with SSRIs, 21 had been treated at outpatient clinics by psychologists/psychiatrists, 3 patients had inpatient treatment stays, and 1 participant had been treated with electroconvulsive therapy (ECT). Three participants were on SSRIs when entering the trial and were included on the terms that they kept their dosage stable throughout the trial.

Diagnostic interviews showed that most participants suffered from recurrent depression (1 mild, 17 moderate, and 8 severe), while the other patients met criteria for a depressive episode (8 moderate and 6 severe). The most common comorbid disorder was generalized anxiety disorder (*n* = 10). Other comorbid disorders included panic disorder (*n* = 2), social phobia, hypochondriasis, eating disorder not otherwise specified, binge eating disorder, and trichotillomania. A total of 13 patients also had comorbid axis II disorders (3 avoidant personality and 10 obsessive compulsive personality disorders). Clusters A and B were exclusion criteria.

**FIGURE 1:**
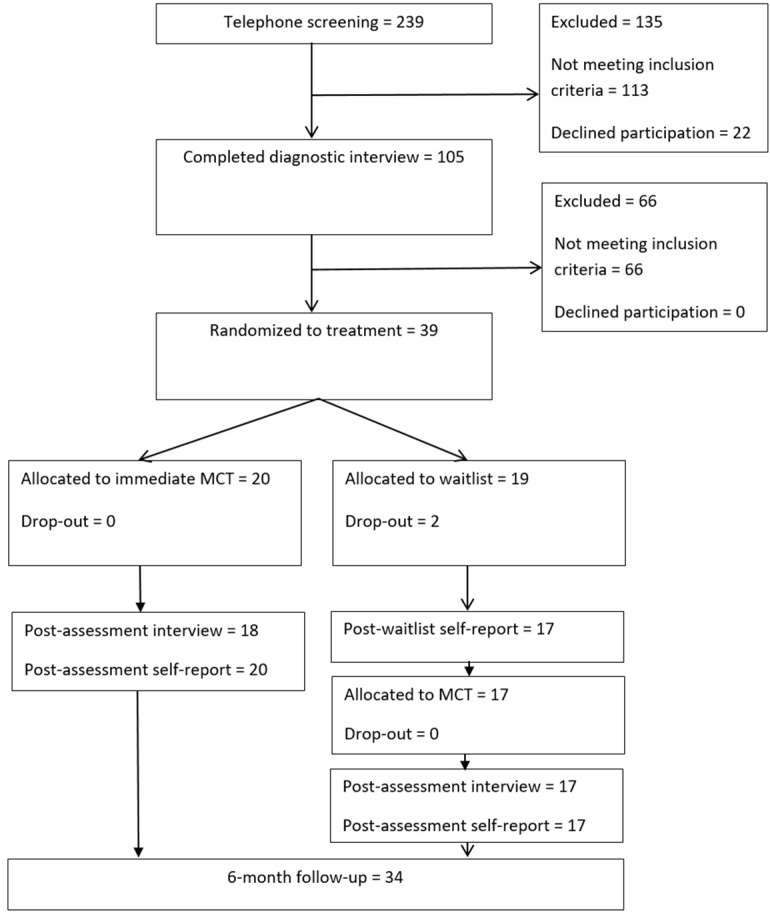
Flow chart.

A description of the participant flow in the study is displayed in **Figure 1**.

### Measures

#### Structured Clinical Interviews

The Structural Clinical Interview for DSM-IV axis I disorders (SCID-I; First et al., 2002) and the Structural Clinical Interview for DSM-IV axis II personality (SCID-II; First et al., 1997) were administered pre- and at post-treatment. SCID-I + II are widely used structured clinical interviews that assess DSM-IV psychiatric diagnoses. After treatment, SCID modules matching the patients’ pre-treatment diagnosis were administered again to evaluate if they still met criteria for clinical diagnosis after receiving treatment.

#### Beck Depression Inventory (BDI; Beck et al., 1961)

The BDI measures levels of depressive symptoms containing 21 self-reported items. Items are rated on a 4-point Likert-scale ranging from 0 to 3, evaluating the severity of each symptom. Several studies have supported the BDI as a reliable and valid measure of severity of depressive symptoms in both clinical and non-clinical populations (Beck et al., 1988). Beck and colleagues have categorized the BDI total scores in the following manner: 0–9 indicates minimal depression, 10–18 mild, 19–29 moderate, and 30–63 indicates severe depression.

#### Inventory of Interpersonal Problems (IIP-64-C; Alden et al., 1990)

In this study, the 64 items version of the IIP with 8 subscales (containing 8 items each) organized in a circumplex manner was used. The eight subscales are labeled: domineering, vindictive, cold, socially avoidant, nonassertive, exploitable, nurturant, and intrusive. The items are scored on a 0 (not at all) to 4 (extremely) scale. The IIP total score is the mean score across all items, representing a global score of interpersonal problems or interpersonal distress. The IIP has received support as a valuable instrument with regard to its sensitivity to change during the course of therapy (Borkovec et al., 2002; Huber et al., 2007). The IIP has also been used to map interpersonal patterns among general outpatient groups (Horowitz et al., 1993; Bjerke et al., 2011) patients with depression (Barrett and Barber, 2007), patients with obsessive compulsive disorder (Solem et al., 2015), and patients with general anxiety disorder (Borkovec et al., 2002).

### Procedure

All subjects gave written informed consent in accordance with the Declaration of Helsinki. The trial was registered at ClinicalTrials.gov^1^ and approved by the Regional Medical Ethics Committee in Norway (ref. nr. 2011/1138). The target group for the trial was patients with a primary depressive disorder.

Inclusion criteria for the study were as follows: (a) signed written informed consent, (b) diagnosed with a primary MDD according to the Structured Clinical Interview for DSM-IV, and (c) 18 years or older. Exclusion criteria were as follows: (a) known somatic diseases, (b) psychosis, (c) current suicide intent, (d) post-traumatic stress disorder, (e) cluster A or cluster B personality disorder, (f) substance dependence, (g) not willing to accept random allocation, and (h) patients not willing to withdraw use of benzodiazepines for a period of 4 weeks prior to entry to the trial.

The recruitment of participants began in January 2013 and ended in January 2015. The trial was advertised through newspapers, radio, social media, and through letters to general practitioners, with information concerning the study and referral. Participants therefore were treatment-seeking individuals referred by their general practitioner or self-referred. Participants were upon contact screened *via* telephone. Possible candidates met with a trained assessor who delivered information about the study, obtained informed consent, and evaluated inclusion and exclusion criteria and severity of depression as well as other psychiatric conditions. Participants were evaluated with SCID-I and SCID-II. An independent assessment team conducted the interviews at pre- and post-treatment. The participants allocated to waitlist also received a telephone call before entering therapy, where the SCID-I depression module was administered by the assessment team. Follow-up data was solely based on self-report. Consensus upon diagnosis was achieved in collaboration with two senior researchers who also watched videotaped recordings of the interviews. Points of assessments were before treatment, after the wait period [waiting list (WL) group only], after treatment, and at 6-month follow-up.

Participants consenting to the terms of the trial, and who met inclusion criteria, were randomly assigned to immediate MCT (10 sessions) or to a 10-week WL. The WL-group received 10 sessions of MCT after the waiting period. Two factors were controlled for in the randomization: gender and number of previous depressive episodes. All participants entering treatment directly after randomization completed treatment. Two participants in the WL group dropped out during the waiting period. The reported reasons were moving away, and starting treatment at a private practice psychologist. These participants did not provide data since pre-treatment, but were still included in the intent to treat analyses. Furthermore, their post-treatment results were replaced using the last observation carried forward method. From the WL condition two patients did not complete all 10 sessions, but terminated treatment after eight and nine sessions. Although these two did not meet with the assessment team for a post-treatment and follow-up interview, their self-report data was available from their latest treatment sessions and used as post-treatment results.

### Treatment

The treatment followed the published manual of MCT for depression (Wells, 2009) consisting of 10 manual-guided sessions. MCT for depression can be summarized briefly in the following way: case conceptualization and socialization followed by (1) increasing meta-awareness by identifying thoughts that act as triggers for rumination, learning about metacognitive control using attention training; (2) challenging beliefs about the uncontrollability of rumination and worry; (3) challenging beliefs about threat monitoring and dangers of rumination and worry; (4) modification of positive beliefs about rumination and worry; and (5) relapse prevention. For a full description of the MCT manual for depression see Wells (2009). There were no specific interventions directed at interpersonal problems.

### Therapists

The therapist group consisted of four clinical psychologists trained in MCT. Supervision was provided by the originator of MCT, by watching videotaped session recordings (translated by the bilingual therapists) and giving ongoing feedback, thus ensuring high implementation quality. In addition, the therapist group met biweekly for peer supervision.

### Data Analysis

The interpersonal profile of our sample, as well as the global amount of interpersonal distress, was explored using descriptive statistics. Correlation analyses were used to investigate the relationship between interpersonal problems and depressive symptoms as well as whether IIP was correlated with depressive symptoms after treatment.

To investigate the effect of MCT on interpersonal problems, a split plot repeated measures ANOVA was used and pre-treatment BDI was entered as a covariate. Effect sizes were calculated using Cohen’s *d* which was based on the average SD from two means. This corrects for dependence between means, using Morris and DeShon’s (2002) equation 8. There were few incidents of missing data (0.4%). In these cases, missing items were replaced using mean item scores on the remaining items.

## Results

### Pre-treatment Characteristics

The IIP total score was not different for patients with or without comorbid axis-I or axis-II diagnoses (*p* = 0.78 and *p* = 0.72, respectively). There were few significant associations with civil status but single patients reported having more problems with being dominant than non-singles. There was no significant gender difference, but a tendency that women rated themselves as more intrusive than men (*p* = 0.051). A total of 93.3% of the sample scored above the suggested clinical cut-off for the IIP-mean score (1.03).

**FIGURE 2:**
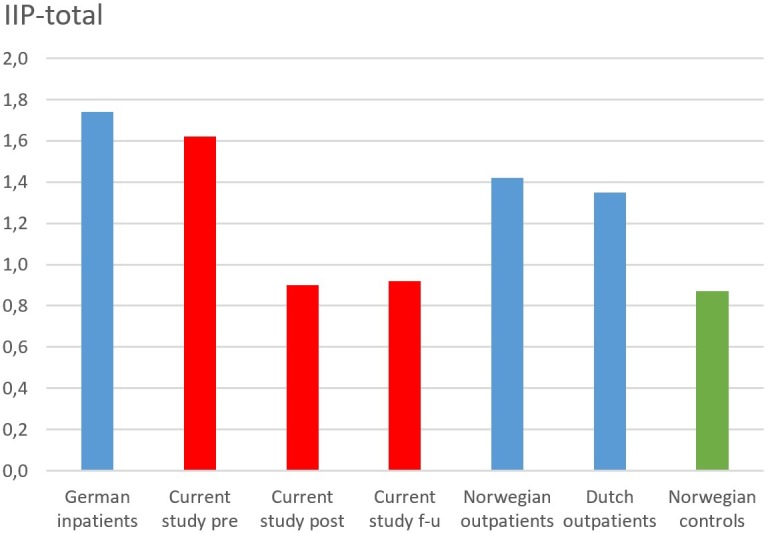
Comparison of studies on pre-treatment levels of interpersonal problems. German inpatients are from Liebherz and Rabung (2014), Norwegian outpatients and controls are from Bjerke et al. (2011), and Dutch outpatients are from Lemmens et al. (2017).

**Figure 2** compares the pre-treatment levels of interpersonal problems in the current study with samples from related studies. Compared with other studies the current sample actually reported more interpersonal problems of this type than other outpatient groups, almost resembling inpatient levels of interpersonal problems. Inpatients at psychiatric clinics from Germany have reported a mean pre-treatment IIP score of 1.74 (*SD* = 0.52) (Liebherz and Rabung, 2014), while depressed Dutch outpatients have reported scores of 1.35 (*SD* = 0.46) (Lemmens et al., 2017). In comparison, the mean score was 1.62 (*SD* = 0.43) in the current study. This suggests that our sample had clear indications of interpersonal problems. The same observation was made in comparison with Norwegian outpatients at psychiatric clinics (*M* = 1.42, *SD* = 0.54) and scores from controls (*M* = 0.97, *SD* = 0.44) (Bjerke et al., 2011). These numbers indicate that the current depressed sample had more interpersonal problems compared to Norwegian controls (*d* = 1.58), outpatients referred to public mental health care (*d* = 0.37), as well as outpatients with obsessive–compulsive disorder (*d* = 0.70) (Solem et al., 2015).

Overall, the comparison showed that the current sample had clearly more interpersonal problems than controls, and possibly more so than other reports of psychiatric outpatients, and close to that of a psychiatric inpatient sample.

### Treatment Effects

Criteria for recovery (Jacobson et al., 1999) from depression involves scoring below 14 and improving at least 9 points on the BDI (Seggar et al., 2002). Using these criteria for the MCT immediate group; 75% were recovered at 6-month follow-up, while 15.0% were improved and 10% showed no change. In the WL condition, 78.9% showed no change, while two had reliable change (10.5%), and one patient recovered. One patient showed signs of deteriorating during the waitlist as BDI scores increased from 24 to 34. At post-treatment, 30.6% had started working or studying, 58.3% were still in work/studies, and 11.1% were still unemployed or on disability benefits.

**FIGURE 3:**
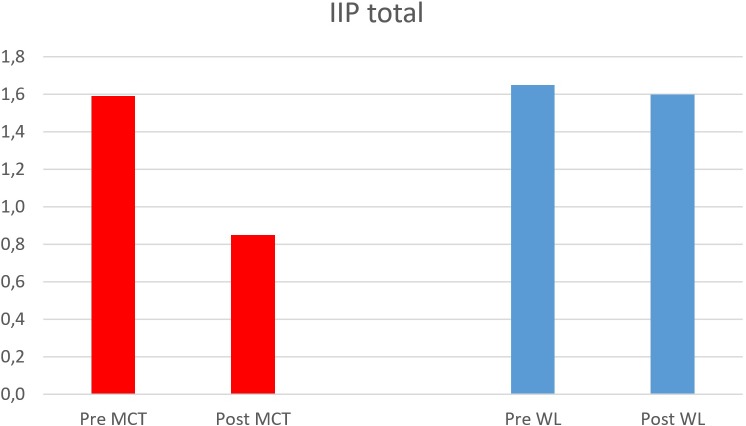
Change in interpersonal problems after waitlist and MCT. MCT, metacognitive therapy; WL, waitlist; Pre, pre-treatment; Post, post-treatment.

As evident from **Figure 3**, patients on the waitlist did not show significant change in interpersonal problems post-waitlist. However, patients assigned to immediate MCT showed large reductions in interpersonal problems.

There were no significant differences between participants in the immediate MCT condition and those in the waitlist condition on pre-treatment IIP, *t*(37) = 0.39, *p* = 0.70, and pre-treatment BDI, *t*(37) = -0.48, *p* = 0.64. A split plot repeated measure ANOVA showed that there was a significant difference in treatment response on the IIP for the waitlist group and the immediate group; time × group, *F*(1, 36) = 17.64 (Pillai’s Trace), *p* < 0.001, partial eta squared = 0.33. The main effect of time across conditions was not significant as participants in the waitlist condition showed few signs of improvement, *F*(1,36) = 3.50, *p* = 0.070, partial eta squared = 0.089. Pre-treatment level of depressive symptoms was entered as a covariate in the analysis but was not significant, *F*(1,36) = 0.71, *p* = 0.41, partial eta squared = 0.019.

For the total sample, 17.9% had a mean IIP score above 2.0 (equal to moderate problems on all 64 items) at pre-treatment. With respect to patients scoring above 2.0 on the respective subscales, the numbers were 76.9% non-assertive, 61.5% for overly accommodating, 59% socially inhibitive, 53.8% self-sacrificing, 20.5% cold, and 18.9% intrusive. Only one patient scored above 2.0 on being dominant 2.6%, likewise with being vindictive. These numbers suggested a friendly-submissive profile for the depressed patients. At post-treatment, these numbers were reduced: 35.9% non-assertiveness, 30.8% overly accommodating, 23.1% socially inhibited, 18.9% intrusive, 15.4% self-sacrificing, 5.1% cold, and none of the patients scored above 2.0 on vindictive or domineering.

As previously mentioned, 93.3% scored cut-off for the IIP-mean score (1.03). At follow-up, that number had been reduced to 43.6%. All of the subscales on the IIP showed significant reductions (*p* < 0.0001) with effect sizes ranging from 0.71 (domineering) to 1.50 (socially avoidant). The effect sizes along with IIP scores at pre- and post-treatment as well as follow-up are further displayed in **Table 1**.

**Table 1:** Scores on IIP-C at pre- and post-treatment as well as 6-month follow-up.

	Pre-treatment		Post-treatment		Follow-up		Pre-follow-up
	*M*	*SD*	*M*	*SD*	*M*	*SD*	*d*
Total	1.62	0.43	0.90	0.66	0.92	0.61	1.36
Vindictive	1.04	0.55	0.51	0.49	0.57	0.50	1.04
Overly accomodating	2.18	0.72	1.35	1.00	1.29	0.93	1.06
Socially inhibited	2.06	0.68	1.01	0.85	0.99	0.82	1.50
Intrusive	1.21	0.57	0.67	0.62	0.73	0.60	0.72
Non-assertive	2.35	0.78	1.30	1.04	1.43	1.03	1.08
Cold	1.39	0.77	0.71	0.74	0.75	0.67	1.05
Domineering	0.81	0.57	0.45	0.47	0.46	0.44	0.71
Self-sacrificing	1.96	0.69	1.21	0.90	1.12	0.85	1.09

*Cohen’s *d* is based on the average SD from two means. This corrects for dependence between means, using Morris and DeShon’s (2002) equation 8*.

### Association Between Interpersonal Problems and Depressive Symptoms

Correlations between IIP scores and BDI scores were analyzed for the three assessment points. At pre-treatment, there were only three significant correlations. Being vindictive, self-sacrificing, and the total score were associated to reporting more depressive symptoms. At post-treatment and follow-up, however, the correlations were stronger, and all but one interpersonal factor (domineering) were significant. A summary of the correlations is displayed in **Table 2**.

**Table 2:** Correlations between interpersonal problems and depressive symptoms.

	Depressive symptoms				
	Pre IIP–pre BDI	Post IIP–post BDI	F-U IIP–F-U BDI	Pre IIP–post BDI	Pre IIP–F-U BDI
IIP total	0.39^∗^	0.62^∗∗^	0.65^∗∗^	0.02	0.01
Vindictive	0.39^∗^	0.60^∗∗^	0.47^∗∗^	0.06	0.03
Overly accomodating	0.23	0.54^∗∗^	0.66^∗∗^	0.01	0.17
Socially inhibited	0.12	0.66^∗∗^	0.60^∗∗^	0.04	-0.02
Intrusive	0.17	0.49^∗∗^	0.47^∗∗^	0.07	0.03
Non-assertive	0.23	0.61^∗∗^	0.58^∗∗^	0.03	0.02
Cold	0.25	0.42^∗∗^	0.40^∗^	-0.07	-0.11
Domineering	0.15	0.31	0.15	-0.04	-0.21
Self-sacrificing	0.39^∗^	0.51^∗∗^	0.72^∗∗^	0.03	0.20

*IIP, Inventory of Interpersonal Problems; Pre, pre-treatment; Post, Post-treatment; F-U, follow-up; BDI, Beck Depression Inventory. ^∗^*p* < 0.05, ^∗∗^*p* < 0.01*.

Correlations also inspected the association between pre-treatment IIP scores and treatment outcome (measured with BDI at post-treatment and follow-up). None of the correlations reached significance. A summary of these correlations is displayed in **Table 2**.

Further analyses revealed that there were no differences in IIP profile at pre-treatment for treatment responders and non-responders (significance values ranged from 0.15 to 0.92). However, treatment responders showed more reduction in interpersonal problems than non-responders. At follow-up, responders had significant lower scores on all IIP subscales, except for domineering (*p* = 0.53). The residual change score correlation for IIP and BDI was 0.75.

## Discussion

Interpersonal problems were clearly present in the current sample of depressed patients and MCT was an effective treatment for these problems. Furthermore, despite previous research suggesting that interpersonal problems could be a predictor of treatment response, this was not the case in the current study. It might be that by not focusing on the problems, but reducing the disorder symptoms and maintaining processing (i.e., discontinuing rumination), the processes were interrupted and maladaptive coping was modified, and as a consequence the patients’ problem focus was diminished, which may include interpersonal problems.

The current sample of depressed patients presented with several interpersonal problems suggesting a friendly submissive style. Compared with other studies, the current sample actually reported more interpersonal problems of this style than other outpatient groups, almost reaching levels of interpersonal problems reported by inpatients. This suggests that at pre-treatment our sample as a group had substantial interpersonal problems.

These interpersonal problems were close to normalized after treatment. The sample only reported slightly more problems with being non-assertive than controls. This suggests a good therapeutic effect of MCT (*d* = 1.36) on interpersonal problems. In comparison, a meta-analysis on short-term psychodynamic psychotherapy for depression reported an effect size of 0.59 (pre- to post) for changes in interpersonal functioning (Driessen et al., 2015). Another meta-analysis of inpatient psychotherapy in Germany found an effect size of 0.35 (Liebherz and Rabung, 2014). Compared to a related study on cognitive therapy (CT) and IPT (Lemmens et al., 2017), the current study presented with a sample which had higher IIP pre-treatment scores and lower follow-up scores. However, these comparisons are not straightforward as patient groups and treatment conditions vary.

As the authors in the CT vs. IPT study discussed, there are fair reasons to believe that CT could lead to changes in interpersonal functioning, both through direct and indirect pathways. Change in interpersonal problems was strongly associated with change in depressive symptoms in the current study. However, this finding does not imply causality as improvement in depressive symptoms could lead to improvement in interpersonal problems and *vice versa*. Scores on theorized mediators are likely to improve over the course of treatment, but this is not necessarily specific for one type of treatment. The current study is the first to show that MCT for depression is also associated with reduction in interpersonal problems. These results could be considered surprising given that MCT does not target interpersonal processes in the same manner or to the same degree as, for instance, (IPT; Weissman et al., 2000).

There are several possible explanations concerning the reduction of interpersonal problems following MCT. The metacognitive model of depression depicts that metacognitive beliefs control, monitor, and appraise thinking. In this model, activation of the CAS can enhance a depressive state which may have interpersonal consequences, or by the selection of maladaptive coping strategies such as social avoidance or reducing activities.

Metacognitive therapy acknowledges the dynamic interplay between an individual and his/her environment. Patients have a tendency to try to avoid exposure to thoughts and events that trigger rumination or worry, which may be related to an experience of reduced executive control. The avoidance may involve various maladaptive coping strategies (e.g., avoid social situations, or people that typically trigger rumination). The maladaptive coping strategies may thus themselves resemble dysfunctional interpersonal factors involved in depression such as submissiveness, passivity, and being withdrawn (Allan and Gilbert, 1997). Another prominent feature of depression is self-criticism, which in MCT theory may be regarded as an example of rumination. Such criticism may contribute to behavioral avoidance, which in turn can contaminate relationships and contribute to interpersonal problems (Fichman et al., 1999).

In MCT, patients are encouraged to abolish maladaptive coping strategies as a response to cognitive events, by replacing the CAS with adaptive coping. Enhancement of self-regulatory capacity, thus strengthening executive control, could then be hypothesized to be beneficial for dealing with interpersonal problems and other external stressors. Future research should therefore investigate the role of dysfunctional metacognitions, because in MCT theory, they are considered the driving forces behind the CAS, hindering self-regulation, which may influence interpersonal functioning and ultimately create problems. It might also be that interpersonal problems are experienced and maintained due to rumination about interpersonal issues. In this case, the problems may dissipate or weaken merely due to less cognitive ruminative maintenance of them. Rather than being adaptive for social problem, solving rumination probably is a maladaptive maintainer of problems (Kennair et al., 2017).

A second explanation concerning the simultaneous effect on depression and interpersonal problems following MCT could be related to MCTs transdiagnostic features in targeting similarities in maladaptive cognitive processing across psychological disorders (Wells and Matthews, 1996; Wells, 2009). The sample had a high level of comorbidity of axis I disorders (with generalized anxiety disorder being the most prevalent) and axis II disorders (with OCPD being the most prevalent). MCT targets repetitive negative thinking, which has been found to be involved across multiple anxiety and depressive disorders, and further significantly elevated in patients with higher levels of comorbidity (Aldao et al., 2010; McEvoy et al., 2013). Patients struggling with interpersonal problems and co-occurring axis I disorders could benefit from therapies targeting essential transdiagnostic mechanisms implicated across psychopathology. Encouraging effects on comorbidity on both axis I and axis II diagnoses have been observed in clinical trials on MCT for depression in cases with high levels of comorbidity (Hagen et al., 2017; Hjemdal et al., 2017). A related line of research also suggests that addressing metacognitions could be beneficial for patients with interpersonal problems (in this case personality disorders). This has been labeled metacognitive IPT (Dimaggio et al., 2017; Gordon-King et al., 2017) and is quite different from MCT, but has adopted some techniques from MCT.

A possible statistical explanation for the large effect sizes observed in the current study could be related not only to the fact that the patients had very high scores on the IIP at pre-treatment but also due to the large change observed in depressive symptoms (Hagen et al., 2017).

Previous findings have indicated that samples with severe interpersonal problems could be more reluctant to change (Muran et al., 1994; Gurtman, 1996; Borkovec et al., 2002), especially if these are related to personality disorder issues. The majority of patients with depression report problems with social avoidance and non-assertiveness before treatment (Renner et al., 2012). Difficulties with being assertive and to subjugate one’s needs have been found to be associated with higher post-treatment depression symptoms (McEvoy et al., 2013). Furthermore, such difficulties could predict poor treatment outcome in patients with generalized anxiety disorder (Borkovec et al., 2002) and depression (Hardy et al., 2001). However, in the current study, there were only non-significant correlations between IIP-scores at pre-treatment and treatment outcome indicating that interpersonal problems were not important in determining the treatment outcome. IIP scores were not related to comorbidity, but there were some associations between interpersonal problems and depressive symptoms. Being vindictive and self-sacrificing were associated with more depressive symptoms at pre-treatment. At post-treatment and follow-up, there were moderate correlations between all subscales (except domineering) and depressive symptoms. The observation that the correlations were stronger post-treatment is probably due to less variance in depressive symptoms at pre-treatment.

This study has different limitations. Measurements of interpersonal problems were solely based on self-report. It is encouraged to use of multiple sources of information when assessing interpersonal problems. Also, global measures of symptomatology and social functioning were not included in the study. However, previous research has suggested that depression and interpersonal problems are consistent predictors of work and social adjustment (e.g., Pedersen et al., 2017). The sample size in the study (*N* = 39) restricted the use of more sophisticated statistical procedures due to lack of statistical strength. Also, it is important to point out that the present study does not draw conclusions about the cause and effect relationships among different variables. Using the BDI to measure symptoms of depression may have yielded slightly different results compared to more recent inventories for depression. However, criteria for recovery are based on using the original BDI, and the BDI has shown to be strongly correlated (0.93) with the BDI-II (e.g., Dozois et al., 1998). Furthermore, a waitlist condition could exaggerate treatment effects and it has been speculated that participants might be more motivated to remain depressed in the wait period. However, observations have also been made that depressive symptoms could be expected to decrease with 10–15% without treatment (Posternak and Miller, 2001) as depression can have a fluctuating course.

Interpersonal problems showed significant and large reductions following MCT for MDD. Furthermore, MCT, which targets established essential transdiagnostic mechanisms across psychopathology, could be a favorable treatment for patients with depression and co-occurring interpersonal problems. Future research should compare MCT with other evidence-based treatments for interpersonal problems related to depression.

## Ethics Statement

All subjects gave written informed consent in accordance with the Declaration of Helsinki. The trial was registered at ClinicalTrials.gov and approved by the Regional Medical Ethics Committee in Norway (ref. no. 2011/1138). The study was conducted without external funding.

## Author Contributions

RH, OH, SS, and LK conducted the therapy in the trial. ES wrote the first draft of the manuscript. All authors have contributed equally in writing up the manuscript.

## Conflict of Interest Statement

The authors declare that the research was conducted in the absence of any commercial or financial relationships that could be construed as a potential conflict of interest.

## Footnotes

1 https://clinicaltrials.gov/show/NCT01608399
